# Monograph on Strabismus

**Published:** 1845-12-01

**Authors:** 


*Monograph on Strabismus, with cases, by Frank H. Hamilton, Professor of Surgery in Geneva Medical College.*—This is a brochure of 72 pages, 18 mo., just issued from the press of Jewett, Thomas & Co. of this city. The first half of the work embraces a concise account of the pathology, etiology, anatomy and the surgical operation. The remainder is devoted to the general results of the operation, and a collection of cases from which these have been deduced. The latter will be found especially valuable. Prof. H. has taken great pains to preserve records of all the cases in which he has operated, and to obtain in each instance, accurate information respecting the permanent result, after a sufficient time has elapsed for this to be determined. The operation for this affection appears to have passed through the two extremes which appertain to the history of most new methods. It was first practised with too much confidence in its general applicability, and is now, perhaps, underrated in this respect. An accurate collection of cases, after the manner which Prof. H. has pursued, will establish its actual value upon a reliable and durable basis. We think the facts presented in this little work will contribute, in a considerable degree, to aid this desirable object.

